# Treatment of subaortic stenosis in hearts with singleventricle physiology

**DOI:** 10.5830/CVJA-2011-023

**Published:** 2012-06

**Authors:** Bulent Saritas, Emre Ozker, Can Vuran, Çağri Gunaydin, Riza Turkoz, Canan Ayabakan

**Affiliations:** Department of Cardiovascular Surgery, Baskent University Hospital, Istanbul, Turkey; Department of Cardiovascular Surgery, Baskent University Hospital, Istanbul, Turkey; Department of Cardiovascular Surgery, Baskent University Hospital, Istanbul, Turkey; Department of Cardiovascular Surgery, Baskent University Hospital, Istanbul, Turkey; Department of Cardiovascular Surgery, Baskent University Hospital, Istanbul, Turkey; Department of Paediatric Cardiology, Baskent University Hospital, Istanbul, Turkey

**Keywords:** pulmonary artery band, univentricular heart, Fontan procedure, subaortic stenosis

## Abstract

**Background:**

We evaluated the patients who had had a Damus-Kaye-Stansel (DKS) operation for single-ventricular physiology with the aorta originating from a hypoplastic ventricle and the pulmonary artery from the systemic ventricle.

**Methods:**

Seven patients who were operated on between May 2007 and November 2010 were evaluated retrospectively. The patients had been diagnosed with a transposed double-inlet left ventricle and triscuspid atresia, and had been waiting for a Fontan operation. Systemic outflow stenosis was defined echocardiographically as those with a gradient greater than 20 mmHg, and angiographically those with greater than 5 mmHg in the subaortic region.

**Results:**

The mean age and weight of the patients was 15 ± 9.7 months and 8 ± 3.3 kg, respectively. The mean gradient between the systemic ventricle and the aorta was 35 ± 25 mmHg. This gradient decreased to 14.3 ± 4 mmHg postoperatively. The early hospital mortality was 14% (one patient). The mean extubation time and mean time in the intensive care unit (ICU) were 13 ± 7.3 hours and 2.2 ± 0.5 days, respectively. The mean follow-up time was 11 ± 2 months. No mortality and semi-lunar valve insufficiency were observed after discharge.

**Conclusions:**

One of the major problems that occur while waiting for a Fontan operation is systemic ventricular hypertrophy and deterioration in the compliance of the ventricle due to systemic ventricular outflow stenosis. When the disadvantages of outflow resection are encountered, a DKS proves to be a good alternative.

## Abstract

In patients with a double-inlet left ventricle (DILV) or tricuspid atresia (TA) where the aorta originates from the hypoplastic ventricle, the interventricular connection that is present at birth may narrow in time.^1^ There is increased pulmonary blood flow in DILV and TA patients with accompanying aortic arch pathology. In order to prevent pulmonary vascular disease, pulmonary artery banding is often preferred as a palliative procedure. However, ventricular hypertrophy caused by the pulmonary band may lead to narrowing of the interventricular connection.^2^-^4^ A restrictive ventricular septal defect (VSD) restricts flow from the systemic ventricle to the aorta, hence leading to progression of subaortic stenosis.

Enlarging the interventricular connection either by resection or by performing a Damus-Kaye-Stansel operation are the two most applied techniques.^5^ We present cases of DILV or TA patients who were found to have subaortic stenosis in their clinical follow up and underwent DKS operations.

## Methods

Seven patients underwent DKS operations between May 2007 and November 2010. These patients had DILV and TA without any chance of bi-ventricular repair and had developed subaortic stenosis while they were waiting for Fontan operations. For the purpose of this study, we defined systemic outflow obstruction as a resting peak instantaneous gradient greater than 20 mmHg on echocardiography or a resting peak-to-peak gradient greater than 5 mmHg with cardiac catheterisation. Systemic outflow obstruction was considered clinically significant if the patient had findings of left ventricular hypertrophy.

Three patients had concomitant bi-directional cava-pulmonary connection (BCPC) operations and one patient had a central shunt operation at the time of the DKS operation. All patients were evaluated in terms of postoperative surgical morbidity and mortality, the degree of subaortic stenosis, ventricle function and rate of re-operation, and semi-lunar valve insufficiency.

## Surgical procedures

*Pulmonary artery banding*: three patients with excessive pulmonary blood flow related to a hypoplastic aorta and the pulmonary artery originating from a non-hypoplastic ventricle underwent a pulmonary artery banding operation through an antero-lateral thoracotomy. One patient also had a Blalock-Hanlon atrial septectomy in addition to pulmonary artery banding, through a median sternotomy. The pulmonary band is tightened until the pressure distal to the band decreases to half of the systemic pressure.

Among these four patients on whom palliative pulmonary band operations were performed, one patient died in the early postoperative period. The other three patients were followed up. The mean age and weight of these four patients was 22 ± 12 days and 3.1 ± 1.9 kg, respectively.

*Bi-directional cava-pulmonary connection*: three patients with balanced pulmonary blood flow underwent BCPC operation. The operations were performed under cardiopulmonary bypass. The main pulmonary arteries were tied up in all patients. The mean age and weight of the patients was 6 ± 2.1 months and 8 ± 3.4 kg, respectively.

*Damus-Kaye-Stansel procedure*: the decision to do a DKS operation was taken for seven patients who had subaortic stenosis in the follow-up period. An 11-day-old patient with pulmonary stenosis and restrictive interventricular connection underwent a DKS and central shunt operation. The procedure was performed under cardiopulmonary bypass and cardiac arrest by the same surgical team. Cardiopulmonary bypass was achieved with aortic and bi-caval cannulation. In patients in whom BCPC operations were planned, the innominate veins were cannulated. In these patients BCPC operations were performed without the use of aortic cross clamping. Cardiac arrest was maintained with antegrade intermittent normothermic blood cardioplegia using the miniplegia technique.

After removing the pulmonary band, which was placed in the initial operation, the pulmonary artery and the aorta were transected. The distal pulmonary artery orifice was closed either with a patch or primarily, according to the orifice diameter. Then the adjacent walls of the pulmonary artery and aorta were joined. The two facing sinuses of both great vessels were sewn together. In order to prevent a mismatch between the diameter of the new artery and the distal aorta, the anterior side of the distal aorta was incised 3 to 4 mm. The new artery was sewn to the distal aorta.

## Results

The demographic data are shown in Table 1. The early hospital mortality was 14% (one patient). After the DKS operation, the pulmonary blood flow was maintained with a BCPC operation in six patients, and with a central shunt in one patient. The 11-day-old patient with transposed TA who underwent DKS and central shunt procedures was taken to ICU with a left ventricular assist device. The patient could not be weaned from high doses of inotropic support and died on the fifth postoperative day due to septicaemia and low cardiac output. The rest of the patients had an uneventful postoperative course. The mean extubation time and stay in ICU were 13 ± 7.3 hours and 2.2 ± 0.5 days, respectively.

**Table 1.** Pre-Operative And Peri-Operative Findings Of The Patients

Age (month)	15 ± 9.7
Weight (kg)	8 ± 3.3
IVC area index (cm^2^/m^2^)	1.88 ± 1.18
Systemic ventricular pressure (mmHg)	132 ± 43
Aortic pressure (mmHg)	91 ± 14
Gradient (mmHg)	35 ± 25
CPB time (min)	113 ± 28
ACC (min)	39 ± 14

IVC: interventricular connection, CPB: cardio-pulmonary bypass, ACC: aortic cross clamp.

The mean duration between the first palliative operation and the DKS operation was 6 ± 1.8 months. There was no statistical difference between patients who had pulmonary banding and patients who had BCPC operations, in terms of timing of the DKS procedure.

When the pulmonary arteries which had been tied up in order to create pulmonary atresia in the initial BCPC operation were transected during the DKS operation, no malformation was observed in the pulmonary valve. However, there was a thrombus on the pulmonary valve in two patients. When the thrombi were removed, the valve structures were found to be normal. All six patients were discharged home on the sixth postoperative day in a good condition.

The mean duration of follow up was 11 ± 1.2 months. There was no mortality in the interim. In routine follow-up echocardiographical measurements, the mean gradient in the systemic ventricular outflow tract was 14.3 ± 4 mmHg. No semilunar valve insufficiency was observed in the follow up. None of the patients was re-hospitalised and none of those awaiting a Fontan operation needed re-operation.

## Discussion

Double-inlet left ventricle and tricuspid atresia with transposed great arteries (TA-TGA) are two forms of a single left ventricle at risk of developing systemic outflow obstruction and poor outcomes.^2^ In these patients, the only way that blood can be delivered to the aorta is through the VSD. Even when the VSD is non-restrictive at birth, it may narrow over time and hence subaortic obstruction becomes apparent.^1^ On the other hand, in patients with distal aortic arch anomalies, pulmonary vascular disease may develop due to the excessive pulmonary flow.^2^,^3^

In these patients, in order to reduce pulmonary blood flow, palliative treatment strategies have been established and pulmonary artery banding is the most common method used. However, since banding induces ventricular hypertrophy, the VSD becomes restrictive and subaortic stenosis develops.^4^ The restrictive VSD limits the flow of blood from the systemic ventricle to the aorta and reduces cardiac output. In addition, ventricular hypertrophy leads to the decrease in the compliance of the systemic ventricle. In patients awaiting a Fontan operation, the ventricle with impaired compliance is unable to maintain the Fontan circulation.^5^

Franklin and colleagues reported 11% survival rate at 10 years in patients with excessive pulmonary blood flow and systemic outflow obstruction. They also reported 79% survival rate at 10 years in patients with pulmonary stenosis without subaortic stenosis.^6^ Therefore, subaortic stenosis should be corrected surgically as early as possible. In order to achieve this goal, the most commonly used methods are BVF resection and a DKS operation.

Direct BVF resection was found to have a high incidence of atrio-ventricular block and high mortality rates.^7^ Lan and colleagues reported 15 complete heart blocks in 44 subaortic resection patients and found that pacemaker requirement and the presence of tachyarrhythmia were important risk factors for mortality.^2^

Postoperative semi-lunar valve insufficiency is the most important disadvantage of the DKS operation. Matitiau and colleagues reported a 10% rate of postoperative semi-lunar valve insufficiency. In our echocardiographical evaluations, we did not detect any semi-lunar valve insufficiency and measured mild gradients between the systemic ventricle and aorta. However our mean duration of follow up was not long enough.

There has been no consensus on the optimal timing of surgery to relieve the systemic outflow obstruction in transposed DILV and TA patients who have developed subaortic stenosis after pulmonary banding. However, Fiore and colleagues recommended performing the operation between the third and sixth month in order to prevent ventricular hypertrophy and related development of compensatory diastolic dysfunction.^1^

In our study group, this interval was approximately six months. This period is appropriate to perform DKS and concomitant BCPC operations, and there is then no need for a second operation to perform a BCPC. Furthermore, in cases where pulmonary blood flow is supplied by systemic-to-pulmonary artery shunt, low diastolic blood pressure and overload may worsen ventricular function.

## Conclusion

In univentricular hearts with narrow interventricular connection, subaortic stenosis increases over time. Relieving the stenosis in the interventricular connection before the dominant ventricle’s function deteriorates is important to do ahead of the Fontan operation. This stenosis can be corrected safely with a DKS operation.
